# Prognostic value of initial recurrence pattern for post-recurrence survival in locally advanced rectal cancer after neoadjuvant chemoradiotherapy and surgery

**DOI:** 10.3389/fonc.2026.1836607

**Published:** 2026-06-30

**Authors:** Chunkang Liu, Jiaolin Zhou, Waiting Lam, Chentong Wang, Junyang Lu, Guole Lin

**Affiliations:** Department of General Surgery, Peking Union Medical College Hospital, Chinese Academy of Medical Sciences & Peking Union Medical College, Beijing, China

**Keywords:** locally advanced rectal cancer, neoadjuvant chemoradiotherapy, post-recurrence survival, recurrence pattern, recurrence-free survival

## Abstract

**Background:**

Postoperative recurrence remains a major cause of treatment failure and death in patients with locally advanced rectal cancer (LARC), despite advances in neoadjuvant chemoradiotherapy (NCRT) and radical surgery. This study evaluated the prognostic value of initial recurrence patterns on post-recurrence survival (PRS) and examined clinicopathological factors associated with recurrence-free survival (RFS).

**Methods:**

This retrospective study included 437 patients with LARC who underwent NCRT followed by surgery. In the cohort without preoperative metastasis, RFS was analyzed using the Kaplan–Meier method and Cox proportional hazards regression models. Among patients who developed postoperative recurrence, initial recurrence patterns were categorized as isolated lung metastasis, isolated liver metastasis, or complex recurrence based on the first site of recurrence. PRS was analyzed with the Kaplan–Meier method, and prognostic factors were identified through Cox proportional hazards regression models. A 2-year landmark analysis was conducted to assess subsequent overall survival (OS) according to recurrence status within 2 years after surgery.

**Results:**

Among the 437 patients, 347 remained recurrence-free, 28 had preoperative metastasis, and 62 developed postoperative recurrence. In the curative-intent cohort, multivariable Cox regression analysis indicated that pathological tumor deposit (pTD) positivity (HR, 2.37; P = 0.006) and ypTNM stage II–III (HR, 1.923; P = 0.012) were independently associated with shorter RFS. Multivariable Cox regression analysis showed that the initial recurrence pattern was independently associated with PRS. Compared with isolated lung metastasis, complex recurrence was consistently associated with poorer PRS, whereas isolated liver metastasis showed an intermediate prognosis. Kaplan–Meier analysis further revealed notable differences in PRS among recurrence patterns, with isolated lung metastasis having the most favorable outcome and complex recurrence the poorest prognosis. Additionally, recurrence within 2 years after surgery was linked to significantly lower subsequent OS in the 2-year landmark analysis.

**Conclusions:**

The initial postoperative recurrence pattern was independently associated with PRS in patients with LARC following NCRT and surgery. Isolated lung metastasis was associated with relatively better outcomes, while complex recurrence indicated a poorer prognosis. In the curative-intent group, higher ypTNM stage and pTD positivity were independently associated with worse RFS. These findings may help improve postoperative risk stratification and support personalized surveillance and management.

## 1 Introduction

Locally advanced rectal cancer (LARC) remains a major challenge in clinical management due to the high risk of local recurrence and distant metastasis, despite continuous advancements in treatment (1). The current standard treatment for LARC involves neoadjuvant chemoradiotherapy (NCRT) followed by total mesorectal excision (TME), which has greatly improved local control and reduced locoregional recurrence rates (2). However, distant metastasis remains the primary cause of treatment failure and cancer-related death (3). Although traditional clinicopathologic factors are commonly used to predict prognosis, their ability to stratify outcomes after recurrence remains uncertain (4).

Recurrence patterns and timing might provide additional prognostic information beyond initial tumor characteristics. Several studies have shown that the biological behavior of recurrent disease, including the initial site of recurrence and the time to recurrence, can influence survival outcomes (5). In particular, early recurrence is often seen as a sign of aggressive tumor biology. However, the prognostic value of initial recurrence patterns and timing in LARC patients treated with NCRT has not been fully elucidated (6). Furthermore, identifying risk factors for postoperative recurrence could help improve treatment strategies and surveillance.

This study extends the current literature by focusing on a relatively homogeneous cohort of patients with locally advanced rectal cancer treated with neoadjuvant chemoradiotherapy followed by surgery. In this setting, we evaluated not only clinicopathological factors associated with recurrence-free survival, but also the prognostic significance of the initial postoperative recurrence pattern for post-recurrence survival, along with the effect of recurrence within 2 years on subsequent overall survival in a landmark framework. These analyses may help improve postoperative risk stratification and support more individualized surveillance strategies.

## 2 Materials and methods

### 2.1 Patients and treatment

This retrospective multicenter study used a prospectively maintained database coordinated by Peking Union Medical College Hospital (PUMCH). Consecutive patients with locally advanced rectal cancer were enrolled from eight tertiary hospitals in China between January 2017 and December 2022. This study was approved by the Institutional Review Board of Peking Union Medical College Hospital (No. JS-1296). De-identified data from participating institutions were collected and analyzed under the coordinating center’s governance framework. The requirement for informed consent was waived due to the study’s retrospective design. All participating centers adhered to standardized criteria for treatment planning, imaging assessment, pathological evaluation, recurrence determination, and follow-up. LARC was defined as clinically staged T3–T4 and/or node-positive disease based on preoperative imaging. All patients received long-course radiotherapy (45–50 Gy in 25 fractions over 5 weeks) concurrently with three cycles of neoadjuvant chemotherapy, administered every three weeks. The chemotherapy regimen included oxaliplatin (85–100 mg/m², IV infusion on Day 1) and capecitabine (825 mg/m², taken orally twice daily on Days 1–14). Radical surgery was scheduled 8 to 10 weeks after completing radiotherapy, and standard adjuvant chemotherapy (ACT) was recommended postoperatively.

**Figure 1:**
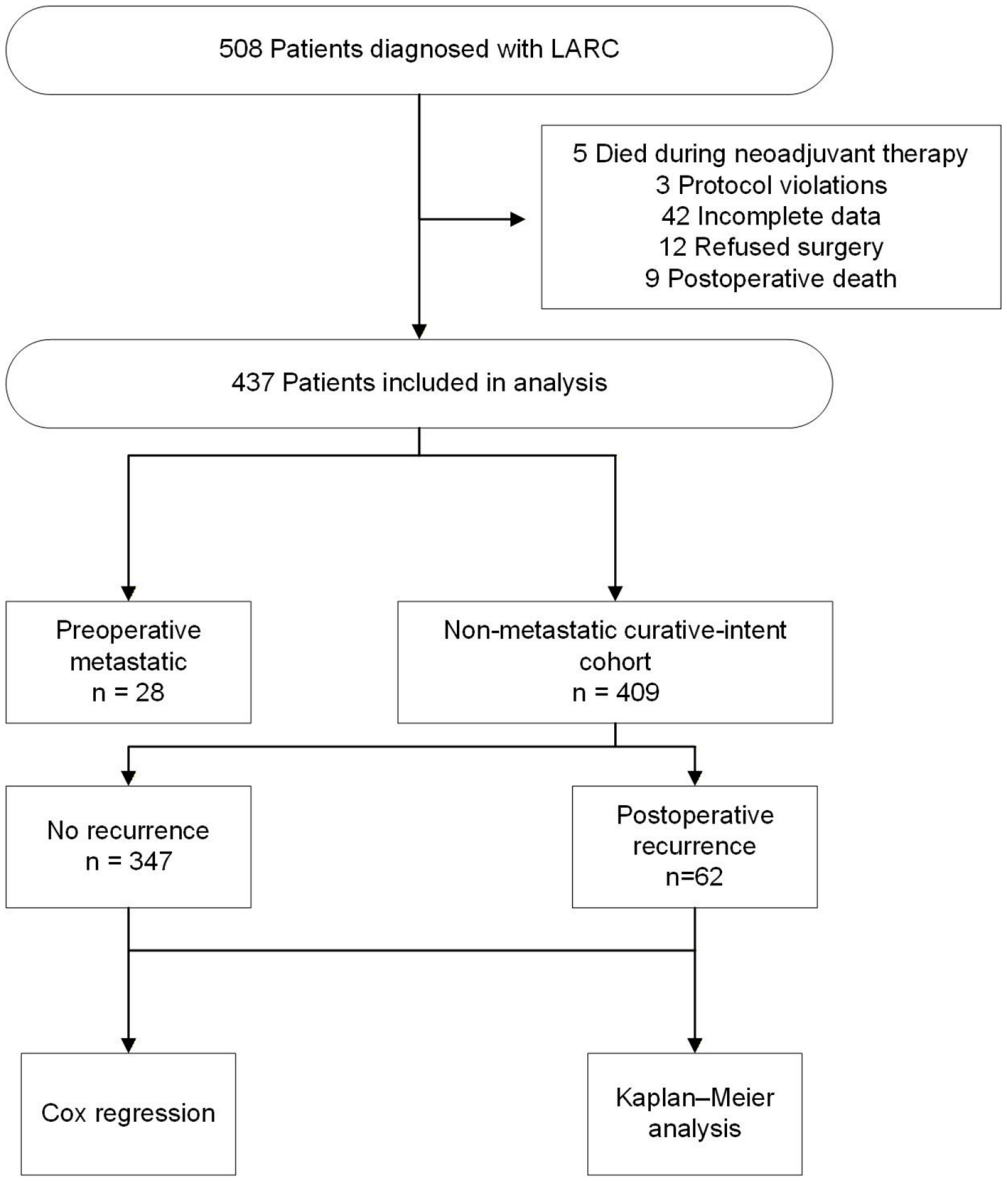
Flowchart of patient selection and study design.

Patients were excluded if they died during NCRT, deviated from the standard treatment plan, refused surgery, died due to postoperative complications, or had incomplete data. Postoperative follow-up included routine physical examinations, serum tumor marker tests, imaging assessments, and colonoscopies when necessary. Overall survival and recurrence status were recorded during long-term follow-up. **Figure 1** presents the flowchart of patient screening, inclusion, and grouping.

### 2.2 Variable definitions

Patients were classified into recurrence-free, preoperative metastatic, and postoperative recurrence groups based on disease status during follow-up. Preoperative distant metastasis was defined as confirmed distant organ involvement through radiology or pathology before radical surgery. Postoperative recurrence refers to the newly detected occurrence of local or distant recurrence after curative-intent resection. Among patients with postoperative recurrence, the initial recurrence pattern was classified by the first documented site of recurrence: isolated lung metastasis, isolated liver metastasis, or complex recurrence. Complex recurrence was defined as recurrence not confined to isolated lung-only or liver-only metastasis, including local recurrence, multiple-organ metastases, peritoneal or nodal metastases, and uncommon distant metastatic sites. This classification was used because isolated lung and isolated liver metastases were the two most common recurrence patterns, whereas other recurrence subtypes were infrequent and not suitable for reliable separate survival analysis. In a secondary exploratory analysis, isolated lung and isolated liver metastases were combined as a single-organ metastasis and compared with complex recurrence. Serum carcinoembryonic antigen (CEA) levels over 5 ng/mL were considered positive, and serum carbohydrate antigen 19-9 (CA19-9) levels above 37 U/mL were also considered positive. pTD was assessed according to the prespecified postoperative pathological reporting requirements in the original prospective multicenter study protocol and were defined as irregular cancer nodules located in the perirectal fat, separated from the primary tumor, and without residual lymph node structure. This definition is consistent with the AJCC TNM concept of tumor deposits. The presence and number of pTD was recorded in the postoperative pathological report. Pathological assessment was performed at each participating center by experienced pathologists according to the prespecified protocol and routine pathological reporting standards. Centralized pathological re-review and formal interobserver agreement testing for pTD diagnosis were not performed.

Early recurrence was defined as recurrence within 2 years after surgery, while late recurrence was defined as recurrence occurring more than 2 years after surgery. A 2-year landmark analysis was conducted, using 2 years after surgery as the landmark time point. To assess the robustness of the 2-year cutoff, exploratory sensitivity landmark analyses were additionally performed using 18-month and 36-month postoperative time points. For each landmark analysis, patients with preoperative metastasis and those who died before the corresponding landmark time were excluded. Subsequent overall survival was calculated from the landmark time point to death or last follow-up and compared according to recurrence status before that landmark time point. Patients with preoperative metastasis and those who died before the landmark time were excluded, resulting in 398 patients who were alive and under follow-up at 2 years after surgery.

For each endpoint, time-to-event was calculated from the date of surgery to the first relevant event or last follow-up. The last follow-up date in this study was November 2025. Recurrence-free survival (RFS) was defined as the interval from surgery to the first documented recurrence or death from any cause, whichever occurred first. Patients who were alive without recurrence were censored at the last follow-up. Overall survival in the landmark analysis was calculated from the 24-month landmark point after surgery to death or the last follow-up. Post-recurrence survival (PRS) was defined as the interval from the first documented recurrence to death or the last follow-up, whichever occurred first. Patients who did not experience the event of interest by the last follow-up were treated as right-censored observations.

### 2.3 Statistical analysis

Continuous variables are expressed as mean ± standard deviation or median (interquartile range), and categorical variables as frequencies and percentages. Group comparisons were conducted using the chi-square test or Fisher’s exact test for categorical variables, and the Student’s t-test or Mann–Whitney U test for continuous variables, as appropriate. In the curative-intent cohort without preoperative metastasis, RFS was estimated using the Kaplan–Meier method and compared using the log-rank test. Univariable and multivariable Cox proportional hazards regression models were used to identify factors associated with RFS. Among patients with postoperative recurrence, the primary survival analysis evaluated PRS according to the prespecified three-category initial recurrence pattern, whereas the comparison between single-organ metastasis and complex recurrence was conducted as a secondary exploratory analysis. PRS was estimated using the Kaplan–Meier method and compared using the log-rank test. Pairwise comparisons among recurrence patterns were adjusted using Bonferroni correction, with a corrected significance threshold of P < 0.017. Cox proportional hazards regression models were used to identify factors independently associated with PRS. Variables with clinical relevance and/or statistical significance in univariable analysis were considered for multivariable modeling. To improve model parsimony and avoid collinearity, variables reflecting overlapping pathological response dimensions were not entered simultaneously into the same multivariable model. Hazard ratios (HRs) with 95% confidence intervals (CIs) were reported. All analyses were performed using SPSS version 25.0 (IBM Corp.), and a two-sided P < 0.05 was considered statistically significant unless otherwise specified. Post-recurrence treatment was summarized descriptively among patients with postoperative recurrence. Because only a small number of patients underwent metastasectomy and detailed information on systemic therapy regimens and treatment lines was not uniformly available across centers, treatment-specific subgroup analyses were not considered statistically reliable. To reduce the potential influence of local radical treatment after recurrence, an exploratory sensitivity analysis excluding patients who underwent metastasectomy was performed.

## 3 Results

### 3.1 Baseline characteristics

A total of 508 patients diagnosed with locally advanced rectal cancer (LARC) were retrospectively reviewed. After excluding patients who died during NCRT, violated the protocol, had incomplete data, declined surgery, or died due to postoperative complications, 437 patients were included in the final analysis. The median follow-up time from surgery was 62 months (IQR, 49–77 months). During follow-up, 347 patients remained recurrence-free, 28 had preoperative metastasis, and 62 experienced postoperative recurrence. Among the 62 patients with postoperative recurrence, isolated lung metastasis was the most common pattern (40.3%, 25/62), followed by isolated liver metastasis (32.3%, 20/62), while complex recurrence was observed in 17 patients (27.4%). The complex recurrence group was heterogeneous, comprising peritoneal metastasis with multiple nodal metastases (n = 3), local recurrence (n = 3), adrenal metastasis accompanied by peritoneal and multiple nodal metastases (n = 2), brain metastasis accompanied by bone metastasis (n = 2), and other multiple-organ metastatic patterns (n = 7). The distribution of post-recurrence treatment is summarized in **Supplementary Table 1**. Metastasectomy was performed in 2 of 25 patients with isolated lung metastasis and in 2 of 20 patients with isolated liver metastasis, whereas the remaining patients received non-surgical intensified therapy after recurrence. Early recurrence (≤2 years) was observed in 47 patients (75.8%), while late recurrence (>2 years) occurred in 15 patients (24.2%).

Baseline characteristics are detailed in **Table 1**. Compared with the recurrence-free group, patients who developed postoperative recurrence more frequently had positive mesorectal fascia involvement (mrMRF), positive extramural vascular invasion (mrEMVI), and a higher circumferential occupation range on preoperative MRI, which was defined as the percentage of rectal wall circumference occupied by the tumor (all P < 0.05).

**Table 1:** Baseline characteristics of patients in the curative-intent cohort according to postoperative recurrence status.

Factors	Recurrence-free n = 347	Postoperative recurrence n = 62	P value
Age, year	59(51,66)	62 (50,68)	0.158
BMI, kg/m²	24.2 (22.1,26.3)	24.4 (21.6, 26.3)	0.987
Gender			
Female	114(32.85)	23(37.10)	0.514
Male	233(67.15)	39(62.90)	
Baseline CEA	3.5(2.1,7.7)	3.6 (1.9,6.4)	0.928
Baseline CA19-9	11.4 (7.0,20.0)	12.1 (8.0,24.9)	0.391
cT stage			
T2	16(4.61)	0(0.00)	0.091
T3	241(69.45)	40(64.52)	
T4	90(25.94)	22(35.48)	
cN stage			
N0	36(10.37)	3(4.84)	0.273
N1	96(27.67)	15(24.19)	
N2	215(61.96)	44(70.97)	
cTNM stage			
II	36(10.37)	3(4.84)	0.172
III	311(89.63)	59(95.16)	
mrMRF			
Positive	148(42.65)	36(58.06)	0.025
Negative	199(57.35)	26(41.94)	
mrEMVI			
Positive	156(44.96)	37(59.68)	0.032
Negative	191(55.04)	25(40.32)	
Perianal muscles			
Negative	307(88.47)	54(87.10)	0.757
Positive	40(11.53)	8(12.22)	
Distance from anal verge, cm	5.7 (4.0,7.7)	5.8 (4.0,7.7)	0.807
Tumor vertical diameter,cm	4.0 (3.3,5.0)	4.5 (3.6,5.4)	0.065
Circumferential occupation range, %	75.0 (50.0,90.0)	77.5 (59.6,100.0)	0.017
Surgical procedure			0.343
Dixon	268(77.23)	45(72.58)	
Miles	54(15.56)	14(22.58)	
Others	25(7.21)	3(4.84)	

Data are presented as median (interquartile range) for continuous variables and n (%) for categorical variables, unless otherwise indicated. Abbreviations: BMI, body mass index; CEA, carcinoembryonic antigen; CA19-9, carbohydrate antigen 19-9; mrMRF, magnetic resonance-detected mesorectal fascia involvement; mrEMVI, magnetic resonance-detected extramural vascular invasion; mrMRF and mrEMVI were assessed on preoperative pelvic MRI.

### 3.2 Cox regression analysis

Univariable and multivariable Cox regression analyses for RFS in the curative-intent cohort are shown in **Table 2**. In the multivariable model, ypTNM stage II–III was independently associated with worse RFS compared with ypTNM stage 0–I (HR, 1.923; 95% CI, 1.154–3.205; P = 0.012), and pTD positivity remained independently associated with inferior RFS (HR, 2.37; 95% CI, 1.287–4.367; P = 0.006). ypEMVI showed a trend toward an association with worse RFS, although the difference did not reach statistical significance (HR, 1.97; 95% CI, 0.954–4.069; P = 0.067). Cox regression analyses for post-recurrence survival (PRS) are shown in **Table 3**. The initial postoperative recurrence pattern was the only factor independently associated with PRS. Compared with isolated lung metastasis, both isolated liver metastasis and complex recurrence were linked to significantly worse PRS. The multivariable Cox model for PRS included baseline CEA, baseline CA19-9, recurrence timing, recurrence pattern, ypTNM stage, and postoperative adjuvant chemotherapy. A total of 35 PRS events were observed; therefore, this model should be interpreted as an exploratory adjusted analysis because of the limited event number and potential risk of overfitting. After adjustment for these variables, the initial recurrence pattern remained independently associated with PRS. To reduce the potential influence of local radical treatment after recurrence, an exploratory sensitivity analysis was performed after excluding the four patients who underwent metastasectomy. In this analysis, complex recurrence remained significantly associated with worse PRS compared with isolated lung metastasis (HR, 4.354; 95% CI, 1.737–10.91; P = 0.002). Isolated liver metastasis also showed a worse survival trend compared with isolated lung metastasis, although the difference did not reach statistical significance (HR, 2.512; 95% CI, 0.927–6.812; P = 0.070). These results were directionally consistent with the primary analysis and are shown in **Supplementary Table 2**.

**Table 2:** Univariable and multivariable Cox regression analyses of factors associated with recurrence-free survival.

Characteristic	Number(%)	Univariate		Multivariate		
		HR (95% CI)	P value	HR (95% CI)		P value
Baseline CA19-9						
Negative	373 (91.2)	1				
Positive	36 (8.8)	1.339 (0.641-2.798)	0.438			
Baseline CEA						
Negative	263 (64.3)	1				
Positive	146 (35.7)	1.098 (0.675-1.787)	0.707			
mrMRF						
Negative	225 (55.0)	1				
Positive	184 (45.0)	1.530 (0.953-2.456)	0.078			
mrEMVI						
Negative	216 (52.8)	1				
Positive	193 (47.2)	1.752 (1.083-2.834)	0.022			
ypTNM						
0- I	233 (57.0)	1		1		
II- III	176 (43.0)	2.387 (1.467-3.886)	P < 0.001	1.923 (1.154-3.205)		0.012
ypEMVI						
Negative	387 (94.6)	1		1		
Positive	22 (5.4)	2.981 (1.479-6.008)	0.002	1.970 (0.954-4.069)		0.067
pTD						
Negative	374 (91.4)	1		1		
Positive	35 (8.6)	3.460 (1.951- 6.136)	P < 0.001	2.370 (1.287-4.367)		0.006

CA19-9, carbohydrate antigen 19-9; CEA,carcinoembryonic antigen; mrMRF, magnetic resonance-detected mesorectal fascia involvement; mrEMVI, magnetic resonance-detected extramural vascular invasion; ypTNM, pathological TNM stage; ypEMVI, pathological extramural vascular invasion; pTD, pathological tumor deposit. mrMRF and mrEMVI were assessed on preoperative pelvic MRI.

**Table 3:** Univariable and multivariable Cox regression analyses of factors associated with PRS among patients with postoperative recurrence.

Characteristic	Number(%)	Univariate		Multivariate	
		HR (95% CI)	P value	HR (95% CI)	P value
Baseline CEA					
Negative	38 (61.3)	1		1	
Positive	24 (38.7)	0.729 (0.354-1.501)	0.391	0.977(0.43-2.219)	0.956
Baseline CA19-9					
Negative	53 (85.5)	1		1	
Positive	9 (14.5)	0.922 (0.353-2.405)	0.868	0.793(0.283-2.216)	0.658
Recurrence timing					
Early recurrence	47 (75.8)	1		1	
Late recurrence	15 (24.2)	1.232 (0.529-2.871)	0.629	1.062(0.425-2.655)	0.898
Recurrence pattern					
Isolated lung metastasis	25 (40.3)	1		1	
Isolated liver metastasis	20 (32.3)	2.627 (1.063-6.489)	0.036	2.691(1.022-7.086)	0.045
Complex recurrence	17 (27.4)	4.437 (1.798-10.945)	0.001	4.949 (1.972-12.42)	0.001
ypTNM					
0-I	23 (37.1)	1		1	
II-III	39 (62.9)	0.733 (0.369-1.455)	0.374	0.711 (0.337-1.503)	0.372
ACT					
No	4 (6.5)	1		1	
Yes	58 (93.5)	1.828(0.249-13.412)	0.533	2.753 (0.357- 21.211)	0.331

CA19-9, carbohydrate antigen 19-9; CEA,carcinoembryonic antigen; ypTNM, pathological TNM stage; ACT, standard adjuvant chemotherapy.

### 3.3 Kaplan-Meier survival analysis

**Figure 2:**
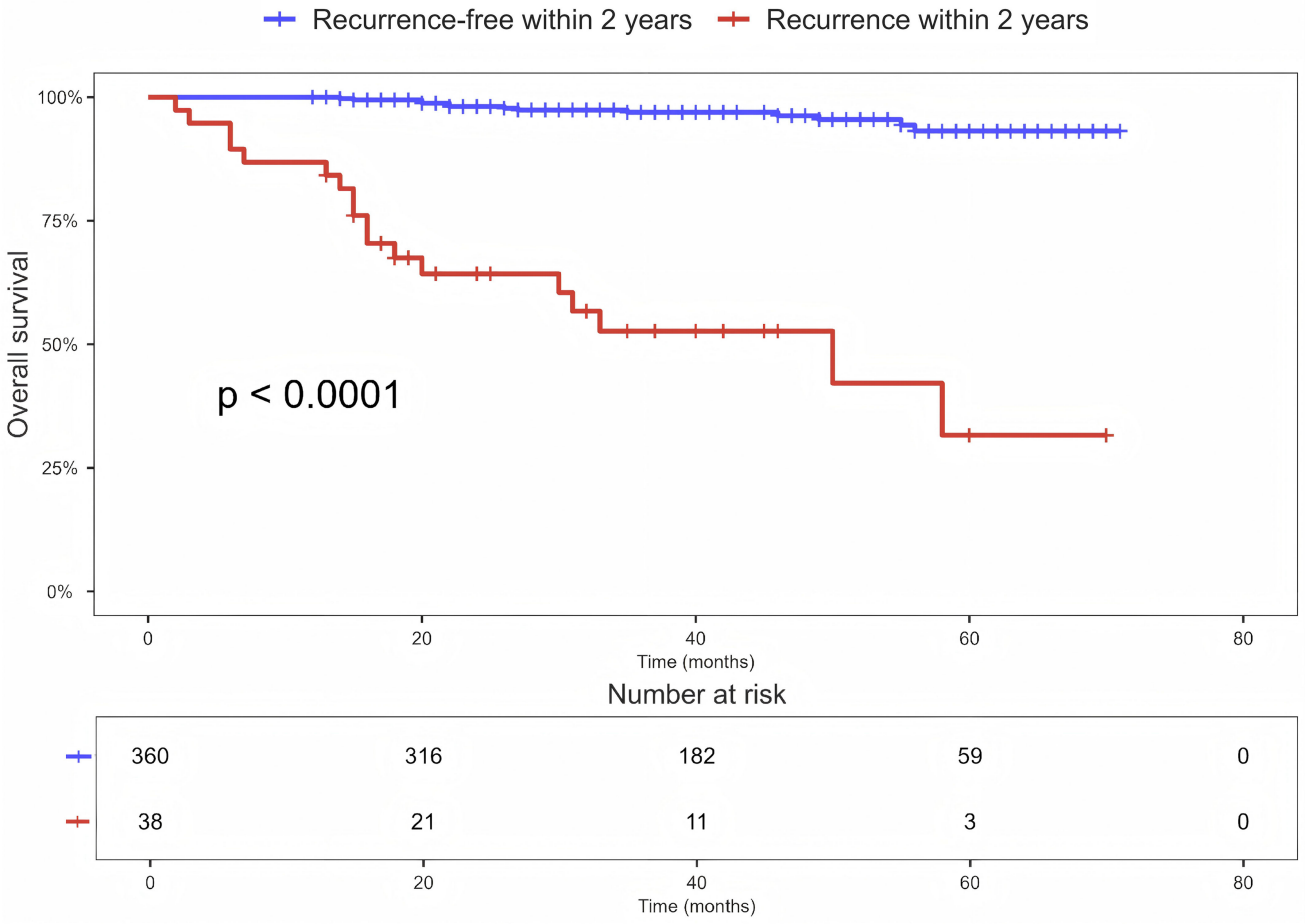
Subsequent overall survival according to recurrence status within 2 years after surgery in the 2-year landmark cohort of patients with locally advanced rectal cancer following neoadjuvant chemoradiotherapy and surgery. Kaplan–Meier analysis revealed that patients with recurrence within 2 years had significantly worse subsequent overall survival than those without recurrence within 2 years after surgery (HR,19.72, 95% CI: 9.61–40.47; log-rank P < 0.001).

**Figure 3:**
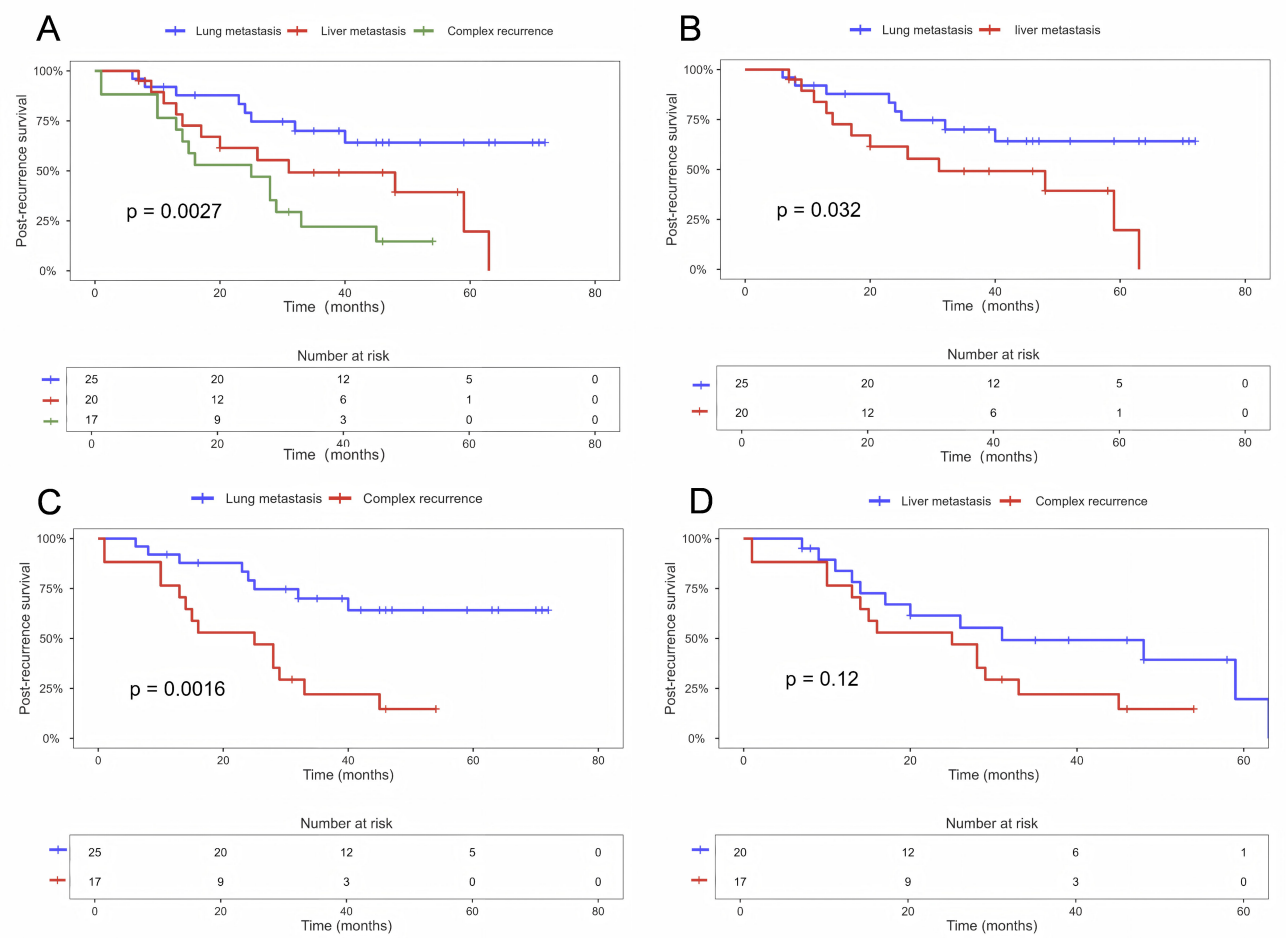
Post-recurrence survival according to initial recurrence pattern in patients with locally advanced rectal cancer following neoadjuvant chemoradiotherapy and surgery. Kaplan–Meier curves comparing isolated lung metastasis, isolated liver metastasis, and complex recurrence. Post-recurrence survival differed significantly among the three recurrence-pattern groups (log-rank P = 0.0027). **(B–D)** Pairwise comparisons of post-recurrence survival between recurrence-pattern groups. Compared with isolated lung metastasis, isolated liver metastasis was associated with worse post-recurrence survival (HR = 2.61, 95% CI: 1.05–6.45; log-rank P = 0.032), and complex recurrence was also associated with worse post-recurrence survival (HR = 1.94, 95% CI: 1.25–3.01; log-rank P = 0.0016). No significant difference was observed between isolated liver metastasis and complex recurrence (HR = 1.92, 95% CI: 0.85–4.37; log-rank P = 0.12). Post-recurrence survival was calculated from the date of first recurrence to death or last follow-up. HRs with 95% CIs were estimated using Cox proportional hazards regression.

**Figure 4:**
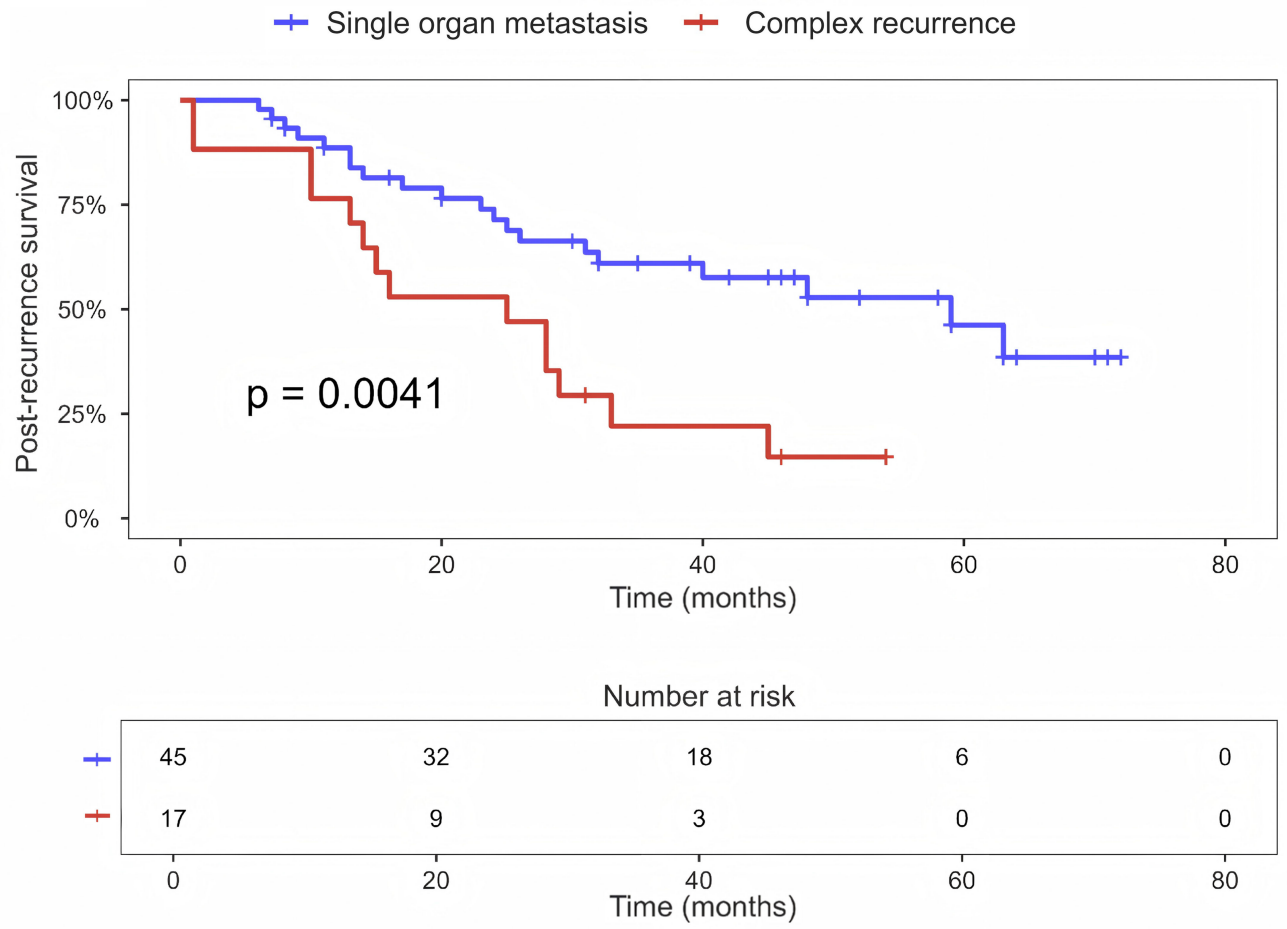
Post-recurrence survival according to complex versus single-organ recurrence in patients with locally advanced rectal cancer following neoadjuvant chemoradiotherapy and surgery. Kaplan–Meier analysis revealed significantly worse post-recurrence survival in patients with complex recurrence compared with those with single-organ recurrence (HR, 2.72; 95% CI, 1.34–5.52; log-rank P = 0.0041).

In the 2-year landmark analysis of patients alive and under follow-up at 2 years after surgery, those who experienced recurrence within 2 years had significantly worse subsequent overall survival than those without recurrence (P < 0.001; **Figure 2**). Exploratory sensitivity landmark analyses using alternative cutoffs showed consistent results. In the 18-month landmark cohort, 400 patients were included, including 28 patients with recurrence within 18 months and 372 patients without recurrence within 18 months. Patients with recurrence within 18 months had significantly worse subsequent OS than those without recurrence (log-rank P < 0.0001; **Supplementary Figure 1**). Similarly, in the 36-month landmark cohort, 393 patients were included, including 40 patients with recurrence within 36 months and 353 patients without recurrence within 36 months. Recurrence within 36 months was also associated with significantly worse subsequent OS (log-rank P < 0.0001; **Supplementary Figure 2**). These findings were directionally consistent with the primary 2-year landmark analysis. PRS differed significantly according to the initial recurrence pattern (P = 0.0027; **Figure 3A**). Patients with isolated lung metastasis had the most favorable prognosis, whereas those with complex recurrence exhibited the poorest outcomes. Pairwise comparisons demonstrated that PRS was significantly lower in patients with complex recurrence than in those with isolated lung metastasis. However, no significant difference in PRS was observed between patients with isolated liver metastasis and those with complex recurrence (**Figures 3B–D**). In a secondary analysis, when isolated lung and isolated liver metastases were combined into a single-organ metastasis group, patients with complex recurrence still had significantly worse PRS than those with single-organ metastasis (P = 0.0041; **Figure 4**). The numbers of events and censored observations in the primary and sensitivity Kaplan–Meier survival comparison groups are summarized in **Supplementary Table 3**.

## 4 Discussion

In this multicenter retrospective study, we found that the initial recurrence pattern was independently associated with post-recurrence survival in patients with LARC who developed recurrence after NCRT and surgery. In addition, recurrence within 2 years after surgery was associated with worse subsequent overall survival in the 2-year landmark analysis. In the curative-intent cohort, higher ypTNM stage and pTD positivity were independently associated with inferior recurrence-free survival.

Previous studies have examined recurrence patterns, early recurrence, pulmonary metastasis, and post-recurrence survival in patients with LARC after neoadjuvant treatment (6–9). These studies have shown that recurrence after NCRT is clinically heterogeneous and that early relapse or specific metastatic sites may influence prognosis. However, relatively few studies have specifically examined post-recurrence survival according to the initial postoperative recurrence pattern in patients treated with standard NCRT followed by surgery. In this context, our study provides an integrated prognostic perspective by demonstrating that the initial recurrence pattern was independently associated with PRS, while also evaluating postoperative pathological risk factors for RFS and recurrence timing using a landmark analysis within the same cohort.

Early recurrence has long been considered an indicator of aggressive tumor biology (10). We selected 2 years as the primary landmark time point because the first two postoperative years are generally considered a high-risk period for recurrence after rectal cancer surgery. However, because there is no universally accepted definition of early recurrence in LARC, we performed additional sensitivity landmark analyses using 18-month and 36-month cutoffs. The results were consistent with the primary 2-year analysis, suggesting that the adverse prognostic impact of early postoperative recurrence was not solely dependent on the selected cutoff. In our cohort, patients who developed recurrence within 2 years after surgery had significantly worse subsequent survival than those who remained recurrence-free during the first 2 years after surgery. This finding aligns with prior studies showing that most colorectal and rectal cancer recurrences occur within the first 2 postoperative years, with early relapse linked to poorer outcomes. Specifically, Shahabi et al. identified a biologically significant cutoff for “early recurrence” at 24–29 months, with patients relapsing within this period exhibiting consistently inferior post-recurrence outcomes (11). Our results support the first 2 postoperative years as a high-risk surveillance period, suggesting that early recurrence may reflect more aggressive tumor biology driven by occult micrometastatic disease not eradicated by multimodal treatment (12). Interestingly, when restricting the analysis to patients who had developed recurrence, early and late recurrences did not significantly differ in post-recurrence survival. This suggests that, once recurrence occurs, the biological behavior and extent of recurrent disease may matter more than the timing of relapse.

In the secondary analysis, patients with complex recurrence had significantly worse post-recurrence survival than those with single-organ metastasis (HR = 2.72, 95% CI: 1.34–5.52, P = 0.0041). This finding suggests that a more extensive recurrence pattern at first detection may reflect both a higher disease burden and a clinically less favorable recurrence phenotype. By contrast, single-organ metastasis may represent a more limited pattern of disease spread, for which local treatment and multidisciplinary management are more feasible. This interpretation is supported by previous studies reporting better outcomes in patients with metastases confined to a single organ, particularly the lung or liver (13). In our cohort, isolated lung metastasis was associated with the most favorable post-recurrence outcome, consistent with reports showing longer survival and slower progression in patients with lung-only metastatic disease (14). Li et al. also showed that lung metastasis progresses more slowly, which is biologically consistent with our findings (9). Taken together, these findings indicate that the initial recurrence pattern provides clinically meaningful prognostic information after relapse and may help identify patients more likely to benefit from more aggressive multidisciplinary management.

Moreover, in the curative-intent cohort, multivariable Cox regression analysis showed that pTD positivity and higher ypTNM stage were independently associated with worse RFS. Residual pathological tumor burden after NCRT remains an important determinant of long-term oncologic outcome, and ypTNM stage provides an integrated assessment of the extent of residual disease after treatment. Several clinicopathological factors have been reported to influence long-term outcomes after neoadjuvant treatment for rectal cancer, but postoperative pathological features may better reflect residual tumor aggressiveness and metastatic potential after treatment (15). In particular, ypTNM stage provides an integrated measure of residual primary tumor extent and nodal involvement after treatment (16), and our results suggest that patients with ypTNM stage II–III remain at substantially increased risk of relapse despite multimodal therapy. Our findings are consistent with those of Baek et al., who showed that, among the NAR score, ypTNM stage, and tumor regression grade, only ypTNM stage remained an independent predictor of disease-free survival after multivariable adjustment (17). Similarly, a higher ypTNM stage was independently associated with worse recurrence-free survival in our cohort, supporting its value as a robust postoperative prognostic indicator in locally advanced rectal cancer. Although tumor deposits are distinct from lymph node metastases, their presence has been linked to a prognosis similar to or even worse than that of nodal disease (18). Zhou et al. pointed out that pTD positivity after NCRT is associated with earlier recurrence, especially within the first two years after surgery, and with a higher likelihood of multiple metastases. Meanwhile, both ypEMVI positivity and pTD positivity were identified as independent prognostic factors for overall survival (OS) (19). Our findings suggest that postoperative pathological factors—particularly residual staging and tumor deposits—may aid in identifying patients at high risk of recurrence following curative-intent treatment. Pathological EMVI and tumor deposits have consistently been associated with adverse oncologic outcomes (20). Although ypEMVI did not reach statistical significance after multivariate adjustment, it still tended to be associated with worse prognosis. pTD positivity may reflect persistent aggressive biological behavior and a greater propensity for recurrence. Although ypEMVI was not statistically significant on its own, the observed adverse trend may still indicate residual invasive potential.

These findings may have practical implications for postoperative surveillance and post-recurrence management. Patients with ypTNM stage II–III disease or pTD positivity may benefit from closer surveillance during the first two postoperative years within existing follow-up frameworks, including more frequent clinical assessment, serum tumor marker monitoring, and chest, abdominal, and pelvic imaging when appropriate (21). Such surveillance may facilitate earlier detection of potentially treatable recurrence and timely multidisciplinary referral. After recurrence is detected, the initial recurrence pattern may help guide treatment triage. Patients with isolated lung or liver metastasis should be considered for early multidisciplinary evaluation of local treatment options, such as metastasectomy, ablation, or stereotactic radiotherapy, combined with systemic therapy when appropriate (22). In contrast, complex recurrence may indicate a higher disease burden and may require more comprehensive systemic management, symptom-directed local treatment, or palliative strategies. However, post-recurrence treatment strategies are not yet standardized, and there are currently no definitive recommendations based solely on recurrence pattern. Therefore, recurrence pattern should be used as a supportive factor for risk stratification and multidisciplinary decision-making rather than as a stand-alone determinant of management. Further prospective multicenter studies are needed to validate these findings and define optimal surveillance and treatment strategies.

This study has important clinical implications because recurrence timing and pattern may help refine postoperative risk assessment. The relatively long follow-up period enabled the evaluation of mid- to long-term survival outcomes and recurrence patterns. Several issues should be considered when interpreting these findings. First, the complex recurrence group was heterogeneous and included local recurrence, peritoneal and/or multiple nodal metastases, multiple-organ metastases, and uncommon distant metastatic sites. Because neoadjuvant chemoradiotherapy followed by total mesorectal excision has improved local control in locally advanced rectal cancer, local recurrence was uncommon in this cohort, with only three patients presenting with local recurrence as the initial recurrence pattern. Therefore, separate survival analysis for local recurrence was not statistically reliable. This classification may have masked prognostic differences among distinct subtypes, and complex recurrence should therefore be interpreted as a pragmatic clinical category rather than a biologically homogeneous entity. Second, post-recurrence treatment was not standardized across centers and may have influenced PRS. Although we performed an exploratory sensitivity analysis excluding patients who underwent metastasectomy, detailed information regarding systemic therapy regimens, treatment lines, treatment response, and other local treatment modalities was not uniformly available. In this sensitivity analysis, complex recurrence remained significantly associated with worse PRS, whereas isolated liver metastasis showed a directionally consistent but non-significant adverse trend. Therefore, residual confounding by treatment heterogeneity cannot be excluded, and the prognostic difference between isolated liver and lung metastasis should be interpreted cautiously. Third, although several baseline characteristics differed between patients with and without postoperative recurrence, this comparison was descriptive and was based on subsequent recurrence status rather than treatment or exposure assignment. Therefore, propensity score matching was not considered appropriate. In addition, the limited number of patients with postoperative recurrence and PRS events precluded reliable matching or extensive covariate adjustment in the PRS analysis, and residual confounding cannot be completely excluded. Fourth, although pTD was assessed according to prespecified pathological reporting requirements, centralized pathological re-review and formal interobserver agreement testing were not performed. Moreover, tumor regression and treatment-related fibrosis after NCRT may complicate the distinction between pTD and completely replaced lymph nodes, vascular invasion, or treatment-related scarring. Therefore, the prognostic value of pTD should be interpreted cautiously and further validated in future studies with centralized pathological review.

## 5 Conclusion

In summary, the initial postoperative recurrence pattern was independently associated with post-recurrence survival, and recurrence within 2 years after surgery was associated with poorer subsequent overall survival. In the curative-intent cohort, higher ypTNM stage and pTD positivity were independently associated with poorer recurrence-free survival. These findings may help improve postoperative risk assessment and support more individualized surveillance strategies.

## Data Availability

The raw data supporting the conclusions of this article will be made available by the authors, without undue reservation.
