# Supplementary figures and images for: Prognostic value of initial recurrence pattern for post-recurrence survival in locally advanced rectal cancer after neoadjuvant chemoradiotherapy and surgery
Source: Front Oncol. 2026 Jun 30;16:1836607. doi: 10.3389/fonc.2026.1836607 (PMC13364688; doi:10.3389/fonc.2026.1836607)

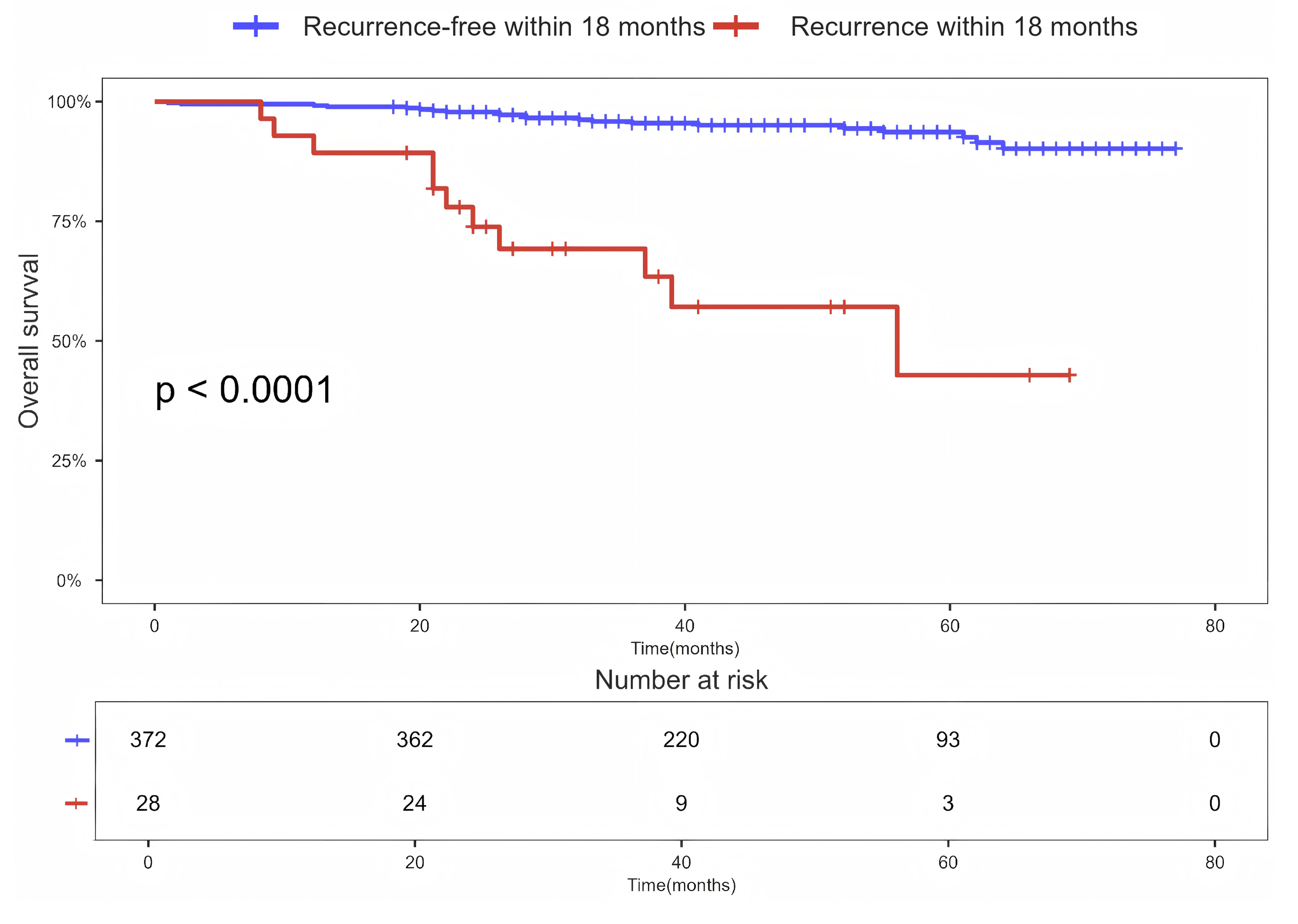

Supplement: Supplementary file 1 [file Image1.jpeg]

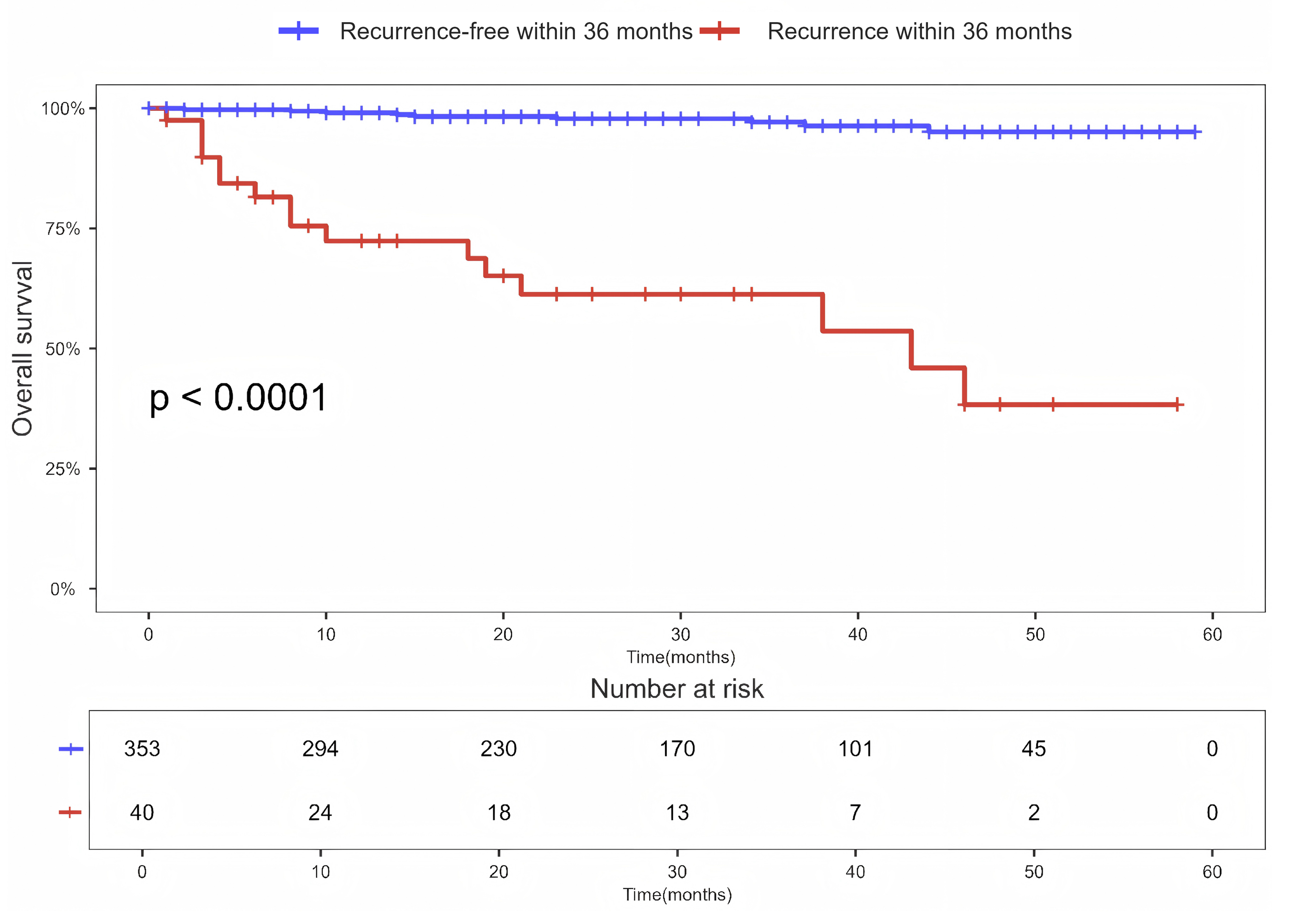

Supplement: Supplementary file 2 [file Image2.jpeg]
