# Supplementary material for: Prognostic value of initial recurrence pattern for post-recurrence survival in locally advanced rectal cancer after neoadjuvant chemoradiotherapy and surgery
Source: Front Oncol. 2026 Jun 30;16:1836607. doi: 10.3389/fonc.2026.1836607 (PMC13364688; doi:10.3389/fonc.2026.1836607)
Supplement: Supplementary file 3 [file Table1.docx]

Supplementary Material

# Supplementary Tables

Supplementary Table S1. Post-recurrence treatment according to the initial recurrence pattern

| **Initial recurrence pattern** | Metastasectomy  (n=4) | Non-surgical intensified therapy  (n=58) |
| --- | --- | --- |
| Isolated lung metastasis  (n=25) | 2 | 23 |
| Isolated liver metastasis  (n=20) | 2 | 18 |
| Complex recurrence  (n=17) | 0 | 17 |

Non-surgical intensified therapy included intensified systemic therapy for isolated lung/liver recurrence and systemic/local multimodal treatment for complex recurrence.
