# Supplementary material for: Prognostic value of initial recurrence pattern for post-recurrence survival in locally advanced rectal cancer after neoadjuvant chemoradiotherapy and surgery
Source: Front Oncol. 2026 Jun 30;16:1836607. doi: 10.3389/fonc.2026.1836607 (PMC13364688; doi:10.3389/fonc.2026.1836607)
Supplement: Supplementary file 4 [file Table2.docx]

| Supplementary Table S2. Univariable and multivariable Cox regression analyses of factors associated with PRS among patients undergoing metastasis resection with postoperative recurrence. | | | | | | | | |
| --- | --- | --- | --- | --- | --- | --- | --- | --- |
| Characteristic | Number(%) | Univariate | | |  | Multivariate | | |
|  |  | HR (95% CI) | | *P* value |  | HR (95% CI) | | *P* value |
| Baseline CEA | |  |  |  |  |  |  |  |
| Negative | 36 (62.1) | 1 | |  |  | 1 | |  |
| Positive | 22 (37.9) | 0.846 (0.407-1.761) | | 0.655 |  | 1.034(0.461-2.321) | | 0.935 |
| Baseline CA19-9 | |  |  |  |  |  |  |  |
| Negative | 49 (84.5)) | 1 | |  |  | 1 | |  |
| Positive | 9 (15.5) | 0.899 (0.342-2.359) | | 0.828 |  | 0.782(0.278-2.202) | | 0.642 |
| Recurrence timing | |  |  |  |  |  |  |  |
| Early recurrence | 44 (75.9) | 1 | |  |  | 1 | |  |
| Late recurrence | 14 (24.1)) | 1.155 (0.467-2.859) | | 0.755 |  | 1.033(0.391-2.73) | | 0.948 |
| Recurrence pattern | |  |  |  |  |  |  |  |
| Isolated lung metastasis | 23 (39.7) | 1 | |  |  | 1 | |  |
| Isolated liver metastasis | 18 (31.0) | 2.320 (0.907-5.931) | | 0.079 |  | 2.513(0.927-6.812) | | 0.07 |
| Complex recurrence | 17 (29.3) | 4.437 (1.798-10.945) | | 0.003 |  | 4.354 (1.737-10.91) | | 0.002 |
| ypTNM |  |  |  |  |  |  |  |  |
| 0-I | 22 (37.9) | 1 | |  |  | 1 | |  |
| II-III | 36 (62.1)) | 0.792 (0.390-1.608) | | 0.518 |  | 0.744 (0.349-1.589) | | 0.372 |
| ACT |  |  | |  |  |  | |  |
| No | 4 (6.9) | 1 | |  |  | 1 | |  |
| Yes | 54 (93.1) | 1.941(0.264-14.259） | | 0.515 |  | 2.895 (0.375-22.379) | | 0.308 |

Supplementary Table S3. Number of events and censored observations in each survival comparison group.

| **Survival analysis** | **Comparison group** | **Total N** | **Events, n** | **Censored, n** |
| --- | --- | --- | --- | --- |
| 2-year landmark OS analysis | No recurrence within 2 years | 360 | 13 | 347 |
| 2-year landmark OS analysis | Recurrence within 2 years | 38 | 18 | 20 |
| 18-month landmark OS sensitivity analysis | No recurrence within 18 months | 372 | 21 | 351 |
| 18-month landmark OS sensitivity analysis | Recurrence within 18 months | 28 | 11 | 17 |
| 36-month landmark OS sensitivity analysis | No recurrence within 36 months | 353 | 9 | 344 |
| 36-month landmark OS sensitivity analysis | Recurrence within 36 months | 40 | 16 | 24 |
| PRS analysis by initial recurrence pattern | Isolated lung metastasis | 25 | 8 | 17 |
| PRS analysis by initial recurrence pattern | Isolated liver metastasis | 20 | 12 | 8 |
| PRS analysis by initial recurrence pattern | Complex recurrence | 17 | 14 | 3 |
| PRS analysis by recurrence extent | Single-organ metastasis | 45 | 20 | 25 |
| PRS analysis by recurrence extent | Complex recurrence | 17 | 14 | 3 |

Note: Events were defined as death for OS and PRS analyses. Patients who did not experience the event of interest by the last follow-up were treated as right-censored observations. OS, overall survival; PRS, post-recurrence survival.

Supplementary Figure S1. Subsequent overall survival according to recurrence status within 18 months after surgery. Overall survival was calculated from the 18-month landmark point to death or last follow-up. Patients with recurrence within 18 months had significantly worse subsequent overall survival than those without recurrence within 18 months after surgery.

Supplementary Figure S2. Subsequent overall survival according to recurrence status within 36 months after surgery. Overall survival was calculated from the 36-month landmark point to death or last follow-up. Patients with recurrence within 36 months had significantly worse subsequent overall survival than those without recurrence within 36 months after surgery.
